# Scimitar syndrome and evolution of managements

**Published:** 2009-12-17

**Authors:** Mahdi Kahrom, Hadi Kahrom

**Affiliations:** 1 Division of Cardiothoracic Surgery, Imam Reza Hospital, Mashhad University of Medical Sciences, Mashhad, Iran.

**Keywords:** Scimitar syndrome, anomalous pulmonary venous drainage, pulmonary venolobar syndrome, Dextrocardia

## Abstract

The Scimitar syndrome is a rare congenital anomaly that consists in part of total or partial anomalous venous drainage of the right lung to the inferior vena cava (IVC). This descending vein is visible on CXR as a curvilinear density along the right heart border and resembles the curved Turkish sword that gives the condition its name. Scimitar syndrome forms part of the large spectrum of associated conditions known as venolobar syndrome. These include right lung hypoplasia or sequestered segments of the right lung, congenital heart disease and various others. Surgical approaches to the Scimitar syndrome have varied according to the anatomic and pathologic features presented in each case. Here we review the clinical signs and symptoms, diagnostic dilemmas, current medical and surgical managements of Scimitar syndrome.

## Background

Scimitar syndrome is a rare association of congenital cardiopulmonary anomalies, and first described by Cooper in 1836 [1,2]. This syndrome is a form of anomalous pulmonary venous drainage (APVD). APVD implies partial or total failure of the pulmonary veins to reach the left atrium. Instead, pulmonary venous drainage is anomalously connected to systemic vein/s, typically to the superior or inferior vena cava (SVC or IVC) or directly to the right atrium (RA). APVD accounts for 2% of all congenital heart disease and is usually associated with left to right shunting at atrial level through some form of atrial septal defect (ASD) [3]. The haemodynamic effects of APVD are also those of left to right shunting at atrial level, thereby exacerbating right heart volume overload. The most common form of partial APVD is connection to the SVC or SVC-RA junction in association with a superior sinus venosus defect. Scimitar syndrome is rarer, with the right pulmonary vein/s draining to the inferior vena cava.

**Figure 1:**
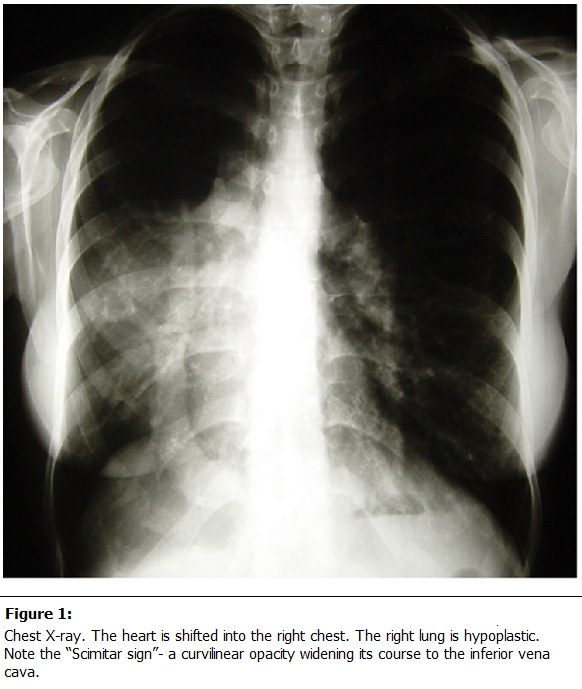
Chest X-ray. The heart is shifted into the right chest. The right lung is hypoplastic. Note the “Scimitar sign”- a curvilinear opacity widening its course to the inferior vena cava.

Associated anomalies are variable and include hypoplasia of the right lung, dextroposition of the heart, hypoplasia of the right pulmonary artery (RPA), and anomalous systemic arterial supply from the aorta to the right lung [4]. The term Scimitar syndrome was coined by Naill in 1960, because of the radiographic appearance of the anomalous vein, which appears as a tubular opacity paralleling the right heart border resembling a curved Turkish sword or Scimitar [5,6]. This is so-called as Scimitar sign ( Figure 1 ).

This rare anomaly has an incidence of approximately 1 to 3 per 100,000 live births [7]; the true incidence may be higher because many patients are asymptomatic. It has been reported most widely in adults and older children and is usually found during a workup for dyspnea, fatigue, recurrent respiratory infections, or as an incidental finding on a routine chest radiograph [7–9]. This adult form of Scimitar syndrome usually is not associated with pulmonary hypertension and typically has mild symptoms and a benign prognosis.

A second, infantile group of patients become symptomatic soon after birth. Their course is often complicated by severe pulmonary hypertension and cardiac failure, making management difficult and mortality high [8–13]. The surgical history of Scimitar syndrome along with current diagnostic strategies, surgical techniques, results, and modalities of follow-up is reviewed.

## Clinical presentation and diagnosis

Scimitar syndrome overlaps with pulmonary sequestration and the term “venolobar syndrome” has been coined to include these associated pulmonary and vascular malformations [14,15]. The condition is associated with [15]:

1. Partial agenesis or hypoplasia of the right lung with bronchial isomerism.
2. Diverticulum or hypoplasia of the right bronchial system.
3. Hypoplasia or agenesis of the right pulmonary artery [16]. This may cause mediastinal shift to the right side and Scimitar vein may be difficult to appreciate or even completely obscured [17].
4. Abnormal systemic blood supply to at least part of the right lung, most frequently the posterior basal segment of the lower lobe, usually arising from the infradiaphragmatic descending aorta [18–22].
5. Dextroposition of the heart due to right lung hypoplasia with mediastinal shift [23].
6. Accessory diaphragm, eventration or partial absence of the diaphragm [24].
7. Phrenic cyst.
8. Horseshoe lung [25,26].
9. Esophageal and gastric lung.
10. Absence of the pericardium.
11. Other congenital cardiac malformations (25% of cases) including ASD [27], ventricular septal defect, coarctation of the aorta, tetralogy of Fallot [28,29], pulmonary stenosis, absent inferior vena cava with azygus continuation to superior vena cava [30,31]. Moreover, the APVD may also be partially obscured, further contributing to pulmonary hypertension [10].

The detection of any of these anomalies should lead to a search for other components. The clinical spectrum of Scimitar syndrome ranges from severely ill infants to asymptomatic adults, and cases may present in one of three ways [11,32]:

- In the neonatal period with respiratory and/or cardiac failure [33]. This is most commonly caused by pulmonary hypertension due to cardiac and/or right lung anomalies. Treatment is surgical and outcome is dependent on the nature and severity of the anomalies [34]. Heart failure may also be caused because of a large arterial supply from the abdominal aorta to a sequestered lobe. In these situations, cardiac catheterization may be used to embolise the aberrant pulmonary blood supply.
- At any stage in life due to recurrent respiratory infections, usually affecting the right lower lobe that often has an abnormal blood supply and venous drainage. Severity and frequency of infections is related to the degree of pulmonary hypoplasia. Lobectomy or even right pneumonectomy may be required to deal with bronchiectasis and prevent further respiratory infections. Affected individuals may also present with haemoptysis due to pulmonary hypertension [35].
- At any stage in life as an incidental finding e.g. due to the detection of a murmur or due to the evident CXR abnormalities [7,36,37].

### Classic findings on physical assessment

Classic findings on physical examination include a shift in heart sounds and cardiac impulse to the right and a systolic murmur. Auscultation of the lung is usually normal, although breath sounds may be diminished on the right. Curiously, Scimitar syndrome is almost exclusively a right-sided anomaly. Only five cases involving the left lung and left pulmonary vein have been reported [38–42].

### Pulmonary hypertension as a marker for Scimitar syndrome

Pulmonary hypertension is often recognized as the cause of the severe symptoms and poor outcome of Scimitar syndrome during infancy [7, 9–11, 43,44]. Multiple factors may be responsible for the pulmonary hypertension. These include the following [7,9,11,41,45,46]:

1. Large left to right shunt via the anomalous pulmonary vein.
2. Left to right shunt from the systemic arterial supply to the right lung [47].
3. Right lung hypoplasia with reduction of the pulmonary vascular bed.
4. Pulmonary vein stenosis and obstruction.
5. Other congenital cardiac malformations.
6. Persistent pulmonary hypertension of the newborn.

Some studies have emphasized that the pulmonary hypertension may be caused by multiple factors including; large left to right shunting by the anomalous pulmonary vein or other intracardiac lesions such as an ASD or VSD [9,13,46]. Pulmonary venous stenosis is a well known cause of pulmonary hypertension in infants with total anomalous pulmonary venous connection and has been recognized as the source of pulmonary hypertension in some patients with Scimitar syndrome [9,12,13,44,48].

## Diagnostic evaluations

The abnormal venous return is the main component of Scimitar syndrome, and gives a characteristic abnormal radiographic shadow descending along the right cardiac border, which resembles a curved Turkish sword (i.e., Scimitar) [49] or a women's leg [50] ( Figure 1 ). However this radiologic sign may be may be obscured because of the associated dextrocardia [7]. Sometimes a “meandering” but normally draining pulmonary vein may be confused for a Scimitar vein [51]. The most striking radiographic feature in each of these patients is the dextroposition of the heart along with varying degrees of opacity of the right hemithorax [4]. A small, opaque hemithorax generally implies volume loss. Mediastinal shift toward the opacified hemithorax with compensatory hyperinflation of the abnormal lung can be seen with atelectasis or pulmonary agenesis [52]. Pulmonary hypoplasia can also cause a unilateral small lung with the heart shifted toward the affected side [53]. The term dextroposition, rather than dextrocardia, is used when the heart is shifted into the right chest but the cardiac chambers maintain a normal relationship and the cardiac apex still points to the left [31].The Scimitar sign is usually absent in infants [11].

Echocardiography may delineate both the Scimitar vein as well as any systemic arterial supply to the right lung [54–55]. Additional cardiovascular anomalies have been noted in 75% of infants with severe symptoms [30]. ASD is the most common associated defect [7,13], but VSD, PDA, tetralogy of Fallot, and left-sided lesions such as aortic arch hypoplasia or coarctation and hypoplastic left heart syndrome have also been reported [8,9,13,30,31]. Fetal echocardiography permits prenatal diagnosis in which spectral and color Doppler provides clues to the presence of an obstructed pulmonary venous pathway [56,57]. Visualization of a confluence behind the right atrium and a vertical vein are the most consistent Echo clues [58].

The flow velocity pattern in the Scimitar vein is different from the normal pulmonary venous flow [59]. The normal flow is biphasic or triphasic, which one or two peaks in systole and one peak in diastole (peak velocity of about 0.5 m/s), and reverse flow at atrial contraction (peak velocity of about 0.2 m/s). The flow pattern in Scimitar vein is monophasic extending throughout the cardiac cycle with no reverse flow at atrial contraction [60].

**Figure 2:**
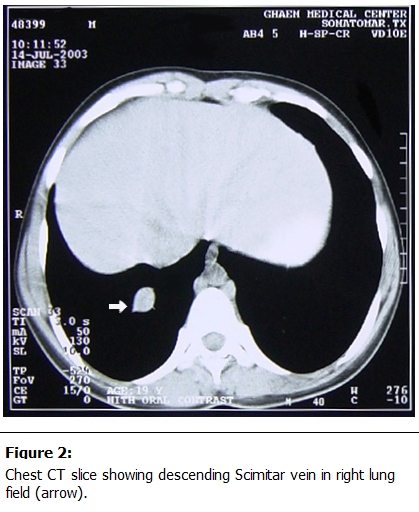
Chest CT slice showing descending Scimitar vein in right lung field (arrow).

Three-dimensional computed tomography (CT) [61,62] and cardiac-gated magnetic resonance imaging (MRI) [63] are useful in visualizing the anomalous pulmonary vein ( Figure 2 ). They can be particularly helpful in detecting an associated horseshoe lung, in which there is posterior fusion of portions of the right and left lungs behind the heart and in front of the esophagus and spine [15,64]. Approximately 80% of infants with horseshoe lung also have Scimitar syndrome [64].

Cine MRI [65,66] and 3-D contrast enhanced MR angiography [63] provides a non-invasive diagnostic technique in the evaluation of anomalous pulmonary venous return. Gadolinium enhanced 3-D MR angiography [67–70] that provides concurrent non-invasive complete anatomical (arterial and venous supply) and functional (calculation of left to right shunt using phase contrast MRI) diagnosis avoids the need for more traditional invasive techniques [71].

**Figure 3:**
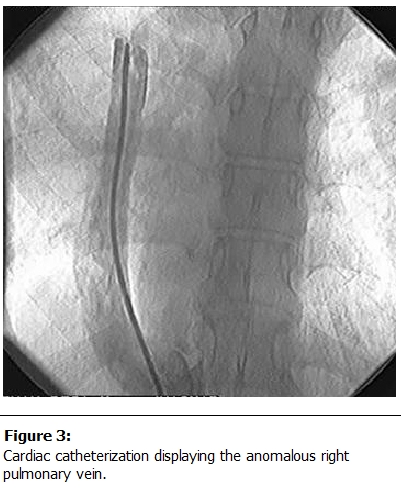
Cardiac catheterization displaying the anomalous right pulmonary vein.

Cardiac catheterization and angiography are probably the most useful procedures for confirming the diagnosis and clarifying the exact anatomy and degree of pulmonary hypertension ( Figure 3 ). Oxygen saturations in the IVC and RA may be increased [7]. The RPA is almost always hypoplastic, atretic, or otherwise abnormal in those infants with pulmonary hypertension [8,42,43,45,72]. An aortogram should also be performed to visualize the presence or absence of an anomalous systemic artery entering the right lower lobe.

## Discussion

Scimitar syndrome is also known as Halasz's syndrome, mirror-image lung syndrome, hypogenetic lung syndrome, epibronchial right pulmonary artery syndrome and vena cava bronchovascular syndrome. It occurs more commonly in females and is occasionally familial [49]. The left lung is very rarely involved and the reason for this, is unknown [38].

The true incidence of this syndrome is unknown since the syndrome may remain undetected in asymptomatic patients who do not undertake a CXR. Patients with Scimitar syndrome may be seen in infancy, childhood or adulthood. Infants typically have features of congestive heart failure from a significant left to right shunt from the anomalous pulmonary venous drainage or more commonly from an additional cardiac defect such as ASD. Older children or adults may have symptoms, and the diagnosis may be made from a chest radiograph that demonstrates the Scimitar sign in as many as 70% of patients, because hypoplasia of the right lung is usually absent in this population. If the atrial septum is open (25%–50% of older patients), examination of the heart may elicit the salient characteristics of an ASD, and the features of Scimitar syndrome will become evident [9]. Functionally, Scimitar syndrome resembles an atrial septal defect (ASD); however, there are no guidelines for surgical correction in adults [73]. Left to right shunt in adults is less than 50% in 82% of patients, pulmonary artery pressures are normal in 77% of patients and slightly elevated in a further 23%, and these patients lead a normal life without surgical correction [7]. However, in cases with a left to right shunt, more than 50% of the patients develop dyspnea, chest infections and pulmonary hypertension [73]. Prognosis after intracardiac repair of Scimitar syndrome is excellent in patients without pulmonary hypertension [74].

The indications for surgical repair include the presence of Scimitar syndrome, especially in association with ASD, pulmonary hypertension, or stenosis of the anomalous vein. Scimitar vein stenosis has been noted in 10% to 20% of cases and in conjunction with the ASD may cause pulmonary hypertension [48]. It is important that the surgical procedure chosen, correct the stenosis in the Scimitar vein; if pulmonary hypertension persists after repair, lung transplantation should be pursued because the long-term mortality among these patients is high.

In neonates and young infants a trial of medical therapy as the initial approach is reasonable to allow an increase in size before repair of the defect. However the presence of pulmonary hypertension or lack of response to medical therapy demands prompt surgical treatment.

The operative approach to correct Scimitar syndrome is quite variable. The first report of surgical therapy for Scimitar syndrome was 1950 by Drake and Lynch [75], who performed a right lower lobectomy. However, efforts should be made to avoid lung resection. Simple ligation of the anomalous pulmonary vein usually results in pulmonary edema, infarction, or both and is not advised.

In 1956, Kirklin et al [76] reported the first total correction without cardiopulmonary bypass in a patient with the infradiaphragmatic type of Scimitar syndrome and an atrial septal defect. Since the vein was not long enough to be implanted directly into the left atrium, it was anastomosed to the right atrium; by means of a Bailey [77] atrioseptostopexy, the flow was directed to the left atrium through the ASD. The experimental basis for this method was established by work of Gerbode and Hultgren [78] who demonstrated in dogs that such an anastomosis would heal well and remain patent. After Kirklin's report, several cases of total correction using open and closed techniques have been reported [79–84].

Several methods with cardiopulmonary bypass have been recommended to repair this anomaly, including direct anastomosis of the Scimitar vein to the left atrium, as reported by Honey [85], or division with reimplantation of the anomalous pulmonary vein into the right atrium with baffle insertion to redirect the flow into the left atrium, as proposed by Shumacker and Judd [86]. Honey suggested that operative management should differ according to the presence or absence of an associated ASD. With an intact atrial septum, the anomalous vein may be reimplanted if possible into the back of the left atrium; otherwise, the anomalous venous return can be baffled through an existing or created ASD to the left atrium [85]. Baffling can be complicated by stenosis resulting in pulmonary venous obstruction [8]. However, successful Inoue balloon percutaneous dilation of a stenotic inferior vena cava anastomosis has been described with good haemodynamic and symptomatic resolution [87].

Alternatively, an intra-atrial patch may be used to create a tunnel, redirecting flow from the anomalous pulmonary vein to the left atrium through an ASD, as described by Zubiate and Kay in 1962 [88]. Puig-Massana and Revuelta [89] described use of free wall of the right atrium to create a tunnel from the Scimitar vein to the left atrium across an ASD. These last two operations require hypothermic circulatory arrest for accurate suturing of the intra-atrial baffle around the orifice of the anomalous vein within the IVC.

There have been two case reports utilizing a 14 mm Dacron graft [90] and a 20 mm Dacron graft [8] interposed between the orifice of the anomalous pulmonary vein and an enlarged ASD as an intra-atrial conduit.

The authors also have an experience in the use of 14 mm hemoshield Dacron graft as an extra-cardiac conduit, between the Scimitar vein and the left atrium, as an alternative approach. Although the fate of the used Dacron grafts in venous positions and their long term patency is not clear, however, in our four year post-op follow-ups by trans-esophageal echocardiography (TEE), the graft is patent and well functioning. Use of anticoagulation therapy is an attempt to maximize the Dacron graft patency [91,92].
